# 320. Differentiating Dengue from COVID-19: A Diagnostic Challenge in the Tropical Regions of the Americas

**DOI:** 10.1093/ofid/ofab466.522

**Published:** 2021-12-04

**Authors:** Fernando Rosso, Olga Lucia agudelo Rojas

**Affiliations:** 1 Fundación Valle del Lili Universidad Icesi, Cali, Valle del Cauca, Colombia; 2 Fundacion Valle del Lili, Cali, Valle del Cauca, Colombia

## Abstract

**Background:**

The differentiation between dengue and coronavirus disease 2019 (COVID-19) diagnoses is a challenge in tropical regions due to the similarity of symptoms and limited access to specific diagnostic tests for each disease. The objective of this study was to describe the initial symptoms and laboratory test values of patients who presented to the emergency department with dengue or COVID-19. A cross-sectional study was performed in a single center in Cali, Colombia

**Methods:**

The inclusion criteria were patients with a diagnosis of dengue or COVID-19 who were older than 14 years of age. All patients experienced fever or other symptoms for fewer than ten days. Linear regression was performed to evaluate the differences in the neutrophil-lymphocyte ratio (NLR) between patients diagnosed with COVID-19 and dengue and was adjusted for sex and age group (≤31 and >31 years). The sample size was calculated to test the hypothesis that the median NLR in COVID-19 patients is higher than that in dengue patients. A p-value < 0.05 was considered statistically significant for all analyses

**Results:**

A total of 93 patients were included: 70 with dengue and 23 with COVID-19. Dengue patients were younger than COVID-19 patients. There were significant differences between dengue and COVID-19 patients regarding platelet count (p< 0.01), neutrophil count (p< 0.01), neutrophil-lymphocyte ratio (NLR) (p< 0.01), and abnormal alanine transaminase (ALT) (p=0.03). The NLR was significantly higher in COVID-19 patients than in dengue patients (p< 0.01).

Table 1. Demographics, clinical and laboratory characteristics in COVID-19 and dengue patients.

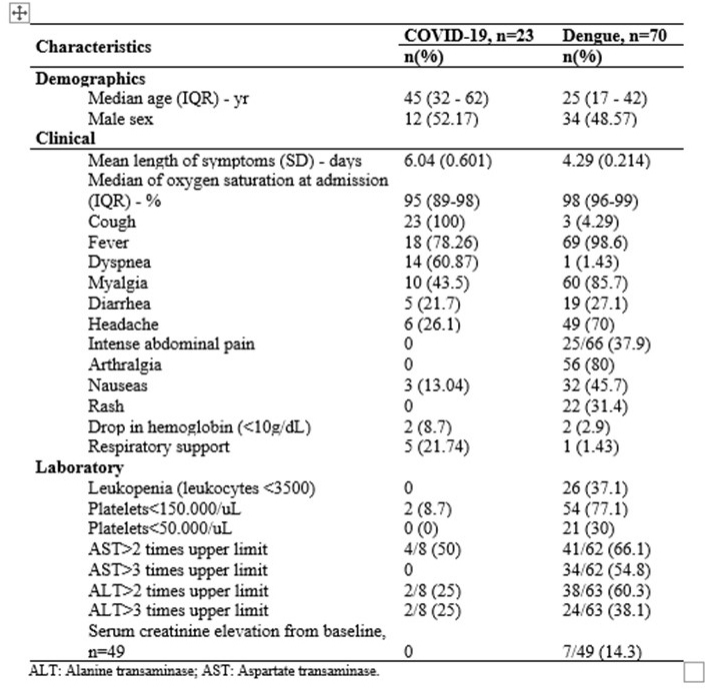

**Conclusion:**

In conclusion, during the first week of symptoms, absolute neutrophil count, NLR, and platelet count could help guide the initial differential approach between dengue and COVID-19. These findings could be useful in geographical areas with a lack of resources.

**Disclosures:**

**All Authors**: No reported disclosures

